# Translation, cultural adaptation and construct validity of the German version of the Adult Social Care Outcomes Toolkit for informal Carers (German ASCOT-Carer)

**DOI:** 10.1007/s11136-020-02682-4

**Published:** 2020-11-02

**Authors:** Birgit Trukeschitz, Assma Hajji, Judith Litschauer, Juliette Malley, Adiam Schoch, Stacey Rand, Ismo Linnosmaa, Julien Forder

**Affiliations:** 1 WU Vienna University of Economics and Business, Vienna, Austria; 2 London School of Economics and Political Science, London, UK; 3 University of Kent, Canterbury, UK; 4 Finnish Institute for Health and Welfare, Helsinki, Finland; 5 University of Eastern Finland, Kuopio, Finland

**Keywords:** Long-term care, Informal care, ASCOT, Cross-cultural adaptation, Validity

## Abstract

**Purpose:**

The Adult Social Care Outcomes Toolkit for Carers (ASCOT-Carer), developed in England, measures the effects of long-term care (LTC) services and carer support on informal carers’ quality of life (QoL). Translations of the ASCOT-Carer into other languages are useful for national and cross-national studies. The aim of this paper was to report on the translation and cultural adaptation of the original English ASCOT-Carer into German, to assess its content validity and to test for its construct validity (convergent and discriminative/known-group validity).

**Methods:**

Translation and cultural adaptation followed the ISPOR TCA guidelines. As part of the translation and adaptation process, five cognitive debriefing interviews with informal carers were used for evaluating linguistic and content validity. In addition, a sample of 344 informal carers of older adults, who received home care services in Austria, was used for hypothesis testing as suggested by the COSMIN checklist to assess convergent and discriminative/known-group validity as part of construct validity.

**Results:**

Cognitive interviews provided evidence that questions and response options of the German ASCOT-Carer were understood as intended. Associations between ASCOT-Carer scores/domains and related outcome measures (convergent validity) and expected groups of informal carers and the care service users they care for (discriminative validity) supported construct validity of the translated instrument.

**Conclusion:**

The German ASCOT-Carer instrument meets the required standards for content and construct validity which supports its usefulness for (cross-)national studies on LTC-service-related QoL-outcomes in informal carers. Research is encouraged to assess further measurement properties of the translated instrument.

**Electronic supplementary material:**

The online version of this article (10.1007/s11136-020-02682-4) contains supplementary material, which is available to authorized users.

## Background

Policymakers have recognized the crucial role of informal carers in supporting older and frail family members, friends, and neighbors and have since implemented benefits to improve their status and well-being [1]. To assess the carers’ situation, a range of instruments [2, 3] has been developed, such as the Carer Experience Scale (CES) [4, 5], the Adult Carers Quality of Life questionnaire (AC-QoL) [6], and the Adult Social Care Outcomes Toolkit for Carers (ASCOT-Carer), all addressing carers’ quality of life, the Zarit Burden Interview [7, 8], addressing care burden. In comparison to other tools assessing carers’ quality of life (QoL), ASCOT-Carer, however, is the only one to have a corresponding instrument for measuring the QoL of long-term care service users [2]. Reflecting both the carers' and service users' situations makes these instruments attractive for a comprehensive assessment of long-term care (LTC) from a QoL perspective.

ASCOT-Carer was developed in England and aims to measure QoL-outcomes of (care) service provision in informal carers [2]. The instrument consists of seven distinct domains to capture areas of adult informal carers’ (family and non-kin unpaid carers) everyday life that may be affected by LTC services and/or services for informal carers [3, 9]. The domains address *Occupation, Control over daily life, Social participation and involvement, Self-care (Looking after yourself), Personal safety, Feeling supported and encouraged, and Space and time to be yourself* (see Table 1). Taken together, these domains form a concept known as “long-term care related quality of life (LTC-QoL)”, also commonly referred to as “social care related quality of life (SCRQoL)” in the English original [9]. A survey instrument that captures the effects of LTC service provision on the caregivers’ QoL contributes to improving empirical evidence and thus to a better understanding of how LTC services meet the needs of informal carers in different aspects of their lives.

**Table 1:** ASCOT-Carer domains. *Source* [3]

Domain	Definition
1. Occupation	Being sufficiently occupied in a range of meaningful, enjoyable activities whether it be formal employment, unpaid work, caring for others or leisure activities
2. Control over daily life	Choosing what to do and when to do it, and having control over their daily life and activities
3. Self-care (Looking after yourself)	Feeling able to look after oneself, in terms of eating well and getting enough sleep
4. Personal safety	Feeling safe and secure, with concerns about safety can include fear of abuse or other physical harm or accidents, which may arise as a result of caring
5. Social participation and involvement	Being content with their social situation, whereby social situation includes the sustenance of meaningful relationships with friends and family, as well as feeling involved and part of their community
6. Space and time to be yourself	Having space and time in everyday life. Enough time away from caring to have a life of their own outside of the caring role
7. Feeling supported and encouraged	Feeling encouraged and supported by professionals, care workers, and others, in their role as a carer

The ASCOT-Carer instruments consist of an interview version with four response levels (INT4) and a self-completion tool with four response levels (SCT4)^1^ which reflect different outcome states: *Ideal state* (3)—individual’s preferences are met, *No needs* (2)—individual’s needs are met but not to the desired level, *Some needs* (1)—there are unmet needs, but no health implications, *High-level needs* (0)—needs have an immediate or longer-term health implication [2, 9]. The total ASCOT-Carer score is the total raw score of the seven domains and ranges from 0 (worst state) to 21 (ideal state). As each domain represents a distinct aspect of the latent construct SCRQoL, weighted combinations of indicators are recommended (Avila et al. 2015). ASCOT-Carer was developed as a preference-weighted measure [9] to reflect the *value* of the care service-induced gain in QoL.

The ASCOT-Carer instrument has gained considerable interest in non-English-speaking countries and has been translated into Japanese, Dutch and Finnish.^2^ The availability of instruments in different languages makes it possible to investigate QoL-effects of LTC service provision on informal carers across nations and within a country among speakers of different languages. A culturally valid adaptation of the ASCOT-Carer instrument can contribute to strengthening the evidence base for policy decisions on how to improve LTC-service provision and the living situation of informal carers. For the translated instrument to be used with confidence, there needs to be cross-cultural equivalence between the valid translated and the original questionnaires [10].

Methodological approaches aiming to establish and evaluate cross-cultural equivalence in questionnaires address both the translation and cultural adaptation process and the assessment of measurement properties of the translated instrument. *Translation and cultural adaptation* (together also referred to as “cross-cultural adaptation”) seek to improve equivalence on a semantic, idiomatic, experiential and conceptual level [11]. For this purpose, systematic multistep guidelines and qualification recommendations for key actors involved in this process have been established (e.g. [11–13]). The assessment of the *measurement model* of the construct is essential as it provides the starting point for the choice of methods to *evaluate the measurement properties* of the culturally adapted instrument. Reflective and formative measurement models differ in terms of the relationship between the construct and its indicators [14] with respect to the nature of the construct, the direction of causality and the characteristics of indicators [15]. In formative models, to which the ASCOT-Carer instruments conceptually belong to, a latent construct (here SCRQoL) is formed by its items (seven ASCOT-Carer domains); variation in the latent construct of SCRQoL is caused by variation in the ASCOT-Carer domains. These seven domains define the construct and are thus not interchangeable, nor do they share a singular common theme. Adding or dropping an ASCOT-Carer domain may change the conceptual meaning of the SCRQoL-construct. In formative measurement models, content and construct validity thus play an important role for assessing validity, defined as the extent to which the interpretation of the results of the measure are warranted [16], at the score and item level. Methods to assess structural validity, such as confirmative factor analysis, and internal consistency, measured by Cronbach’s alpha, are appropriate for reflective, but not for formative models, in which items may correlate positively, negatively or not at all [14].

The aim of this study was to translate and culturally adapt the original English-language ASCOT-Carer instruments (INT4 and SCT4) into German and to examine aspects of validity of the translated instrument. We report on the results of assessing linguistic and content validity as part of the translation and adaptation process. In addition, we investigate construct validity of the translated measure using survey data for testing expected relationships of the German ASCOT-Carer instrument with comparator outcome measures (convergent validity) and with selected subgroup characteristics of informal carers (discriminative or known-groups validity). As the ASCOT-Carer instrument follows a formative model, each domain is relevant for defining the latent construct. Thus, we assess construct validity on both the score and item level of the translated instrument. We shed light on challenges for cultural adaption and validation and discuss solutions to these challenges that may inform future studies on adapting the English ASCOT-Carer instrument into other languages. A valid German version of the ASCOT-Carer instruments will be a useful tool for national and cross-national surveys on the effects of LTC services on the QoL of German speaking informal carers.

The rest of the paper is organized as follows: First, the methods section describes the approaches applied for cultural adaption, the methods for assessing linguistic, content and construct validity. Then, results of the current study are presented, followed by a discussion of the main findings and comparison to previous work on validation of the original English ASCOT-Carer.

## Methods

### ASCOT-Carer translation into German and cultural adaption

The ASCOT-Carer instruments (INT4, SCT4) were translated from English into German between June 2015 and March 2016 by the Austrian research team in cooperation with the translation agency PharmaQuest (now part of Corporate Translations, Inc.) and the English ASCOT development team. In line with ISPOR’s principles of good practice for the translation and cultural adaptation (TCA) process for patient-reported outcome (PRO) measures [12], key actors and methods were chosen to ensure appropriate cross-cultural equivalence of the English and German ASCOT-Carer. The translation company involved four bilingual translators, an in-country investigator familiar with the country’s care practice, and one independent proofreader. The Austrian research team had considerable experience with ASCOT as well as with care policy and practice in German speaking countries. The ASCOT development team was previously involved in the cultural adaption of ASCOT (service user measure) into other languages.

**Fig. 1:**
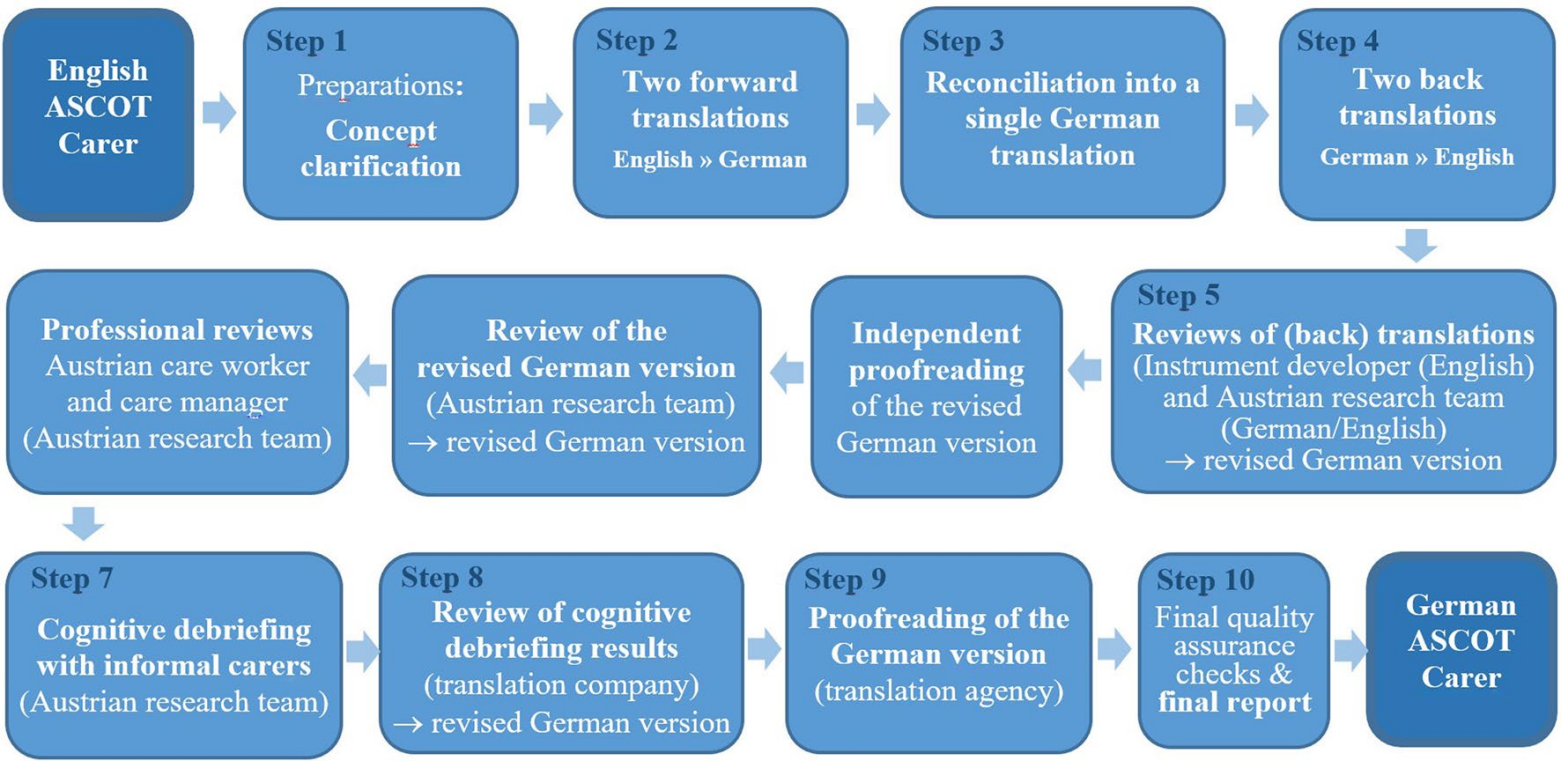
Flow diagram of the translation and cross-cultural adaptation process of ASCOT-Carer (English into German) following ISPOR TCA. Notes: Step 6—harmonization of new translations (in our case with the Finnish translation)—was part of key steps 5 and 7. Source: PharmaQuest Ltd. [17], authors' illustration

Figure 1 describes the steps of the translation and cross-cultural adaptation process [17] following Wild et al. [11]. The ASCOT-Carer concept clarification guide, previously produced by the translation company and approved by ASCOT development team, defined the conceptual meaning of each item and its response options of the ASCOT-Carer measures (Step 1). Based on this guidance, translators, native in German and fluent in English, drafted two initial translations (Step 2). These were then reconciled into a single version by an in-country investigator who held a degree in translation and interpreting and had worked as a nurse (Step 3). The revised version was back-translated into English independently by two translators, native in English and fluent in German (Step 4) for further review by the translation agency’s in-country investigator, the ASCOT development team and the German-speaking research team (Step 5). Step 6 of the ISPOR TCA guideline addresses the process of harmonization which aims to consider all new translations (into different languages). As at the time of translation German and Finnish versions were the first ASCOT-Carer instruments to be translated, the Austrian and Finnish teams shared and discussed their results at key steps 5 and 7 of the adaptation process. Going beyond the ISPOR TCA guidelines, the revised German version was then proofread by an independent translator, not previously involved in the translation process, and reviewed by the Austrian research team, who also conducted professional reviews with an Austrian care worker and a care manager. Then, cognitive debriefings with five informal carers were carried out by the Austrian research team (Step 7) and comments were sent to the translation agency. The translation agency reviewed the results of the cognitive debriefing (Step 8) and made amendments where necessary. The pre-final version was proofread (Step 9) by the in-country investigator before being approved by the translation agency (Step 10). The original English version and the final German translation of the ASCOT-Carer instruments (INT4 and SCT4) are available on the ASCOT website (www.pssru.ac.uk/ascot or https://short.wu.ac.at/ascot).

### Data collection

We used two data sources to improve cultural adaptation and to assess the construct validity of the German version of the ASCOT interview for informal carers (ASCOT-Carer INT4): Cognitive debriefings as part of the translation and adaptation process aimed to give insight into the understanding of question and response option intent and the meaning of terms to the respondents. Two researchers (one woman, one man), trained in conducting cognitive interviews with older people, were provided with a comprehensive interview guide that comprised general instructions for the interviewers as well as tasks and questions for the interviewees right after each ASCOT-Carer item and the related set of response options. All cognitive debriefing interviews were recorded. After each interview, experiences and first results were reflected on in the Austrian research team. As per the ISPOR’s TCA group [11] and the translation agency’s recommendations [17], five face-to-face cognitive interviews were carried out in November and December 2015, with the possibility of increasing the sample size if needed. As the initial analysis of the transcripts showed a great deal of overlap in how the respondents understood the items and little variation in terms of the types of misunderstandings, no further interviews were carried out. The respondents were informal carers whose relatives received home care services from care service providers in Vienna. Each respondent was asked to fill out a copy of the whole translated questionnaire, to comment on the wording of questions and response options that were difficult to understand, and to suggest alternative wordings throughout the process. The cognitive debriefings with informal carers combined the think-aloud method with verbal probing techniques [18].

In addition, cross-sectional quantitative data on informal carers of home care service users were collected via a survey in 2016/17 as part of the EXCELC project in Austria. In a first step, home care service users in all nine Austrian provinces were recruited for study participation in cooperation with the Federal Ministry of Labor, Social Affairs and Consumer Protection as well as local authorities and care organizations. Service users who reported receiving informal help were asked to provide contact information for their main informal carer, who was then interviewed face-to-face using a standardized questionnaire. In total, 344 informal carers of LTC service users were interviewed across all nine Austrian regions (Laender) using computer-aided personal standardized interviews (CAPI) designed by the online survey software ‘Qualtrics’.^3^ The interviewers used showcards for the ASCOT-Carer section in the questionnaire. Details on the data collection are reported elsewhere [19].

### Assessing linguistic and content validity of the German ASCOT-Carer

Cognitive interview transcripts were used to assess linguistic and content validity to inform changes needed to further improve the cross-cultural adaptation of the translated ASCOT-Carer. *Linguistic validity* ensures the conceptual and linguistic equivalence between the original and translated version of the instrument [20]. An item-based analysis was performed in order to identify discrepancies in meaning between the original and translated versions of ASCOT-Carer items or response options. It consisted of summarizing responses, identifying problem areas and proposing suggestions and amendments where needed [21]. *Content validity* refers to how well an instrument’s content reflects the constructs to be measured [22] and whether it is comprehensive, understandable and acceptable [23]. Cognitive interview transcripts and the ASCOT concept clarification guide [9, 12] were used to assess whether the translation adequately reflected the original content of the ASCOT items and response choices.

### Assessing construct validity of the German ASCOT-Carer

Survey data were taken to assess the construct validity of the German translation of the ASCOT-Carer instrument. The COSMIN (COnsensus-based Standards for the selection of health Measurement INstruments) checklist [24] recommends hypotheses testing for investigating construct validity (box 9a+b) of the translated instrument [24, 25]. We performed a series of bivariate tests of association to examine expected relationships between the ASCOT-Carer measure with comparator outcome measurement instruments to assess convergent validity and with selected subgroups of informal carers to evaluate discriminative or known-groups validity. A complete overview of the measures expected to be related with the German version of the ASCOT-Carer instrument is given in Table 8 in the Online Appendix.

Based on theoretical considerations or previous empirical evidence, we expected the *overall ASCOT-Carer score* to be related with scores of comparator outcome measures, such as QoL and health-related QoL as well as indicators of care experience and care burden [3]. The variables used for testing the hypothesized relationships include self-perceived quality of life (QoL) as a seven point measure (seven indicating the highest possible QoL), and health-related QoL, measured by EQ-5D-3L items (high values indicating better states) and the EQ-5D index (anchored at 0 for death and 1 for perfect health using German weights [26, 27]). As indicators of care burden, we used the Carer Experience Scale (CES) [4] (ranging from 0 to 18, with high values indicating better states), the Zarit Burden Interview (ZBI) using the four-item screening version [28] (ranging from 0 to 14, with high values indicating high caregiving burden), questions on service users’ cognitive performance and behavior and care intensity measured in hours. To evaluate the validity of the *individual ASCOT-Carer domains*, we used items of the outcome measures that are expected to reflect the individual domains of the translated measure. For the *comparison between subgroups of informal carers*, we compared informal carers with high and low care intensity (measured by care hours per week), carers’ opportunity of getting break from caring or not and informal carers who cared for relatives or non-kin with/without cognitive skills or challenging behavior. We also used selected items of comparator outcome measures (selected item of EQ-5D and ZBI) to form groups of carers to investigate the relationships with selected ASCOT-Carer domains.

For ASCOT-Carer scores related to comparative outcome scores, we used Spearman’s rho to assess correlations. Thresholds for association effect sizes were chosen according to Cohen [29]. Fisher’s exact tests (for categorical variables) and one-way analyses of variance, ANOVA, (for continuous variables) were used to test the hypothesized associations for ASCOT-Carer domains. Benjamini and Hochberg [30] correction for multiple testing was applied. For ANOVA results, omega squared was chosen to assess effect size as it is less biased then eta squared in smaller samples [31]. For the thresholds see Cohen [29]. Convergent validity is perceived as adequate if more than 75% of the expected relationships can be supported by the analysis results or if a correlation between the measures expected to be related is stronger than 0.5 [32]. All statistical analyses were conducted in Stata v15 [33].

## Results part 1

### Cultural adaptation during the translation process

Some sections of the translation of the ASCOT-Carer instruments into German required cultural adaptation to appropriately reflect the meaning for the German-speaking target group. First, a few English expressions challenged the forward translation into German (e.g. “control over daily life”, “to feel clean and presentable”, “thinking about myself”, “to be yourself in your daily life”) as there was no meaningful direct translation. Where no appropriate final wording was found in the translation process, alternative expressions were tested in the professional review and cognitive debriefing phases (e.g. for “control over daily life”). Second, we found that some translations into German resulted into back translations that did not literally match the original because of idiomatic expressions which were preferred to literal translations (e.g. ‘role’ as a carer was changed to ‘task’ of a carer). Third, contemporary German language seeks to account for the gender of the actors, e.g. ‘der Interviewer’ (male interviewer), ‘die Interviewerin’ (female interviewer). As English usually has no grammatical gender, the gender of German nouns (masculine, feminine or neutral) added complexity to sentences referring to actors. Last but not least, cultural adaptation was needed for the terms referring to specific actors (e.g. carers), services and institutions (e.g. the British National Health Service) in the English and Austrian long-term care systems.

### Linguistic and content validity of the German ASCOT-Carer

Linguistic and content validity were also evaluated by cognitive debriefing interviews. Five female informal carers, aged 46–72, caring for their relatives between one and 20 years, were involved in the cognitive debriefing to assess how well questions and response options were understood and whether the wording was appropriate.

The cognitive interviews showed that the respondents’ comprehension of the German translation of the ASCOT-Carer instrument was satisfactory, as they were able to adequately explain their responses with respect to each of the ASCOT-Carer domains. Minor changes to the wording were made in cases where at least one interviewee expressed confusion or ambiguity or misunderstood a part of the item (e.g. the preferred translation for ‘support’ was ‘Unterstützung’ (assistance), not ‘Hilfe’ (help)). These changes to the translation were made in accordance with the English ASCOT development team. Respondents generally understood the four response options as intended and were able to distinguish between them without difficulties. We used different German expressions (‘wie ich es will/möchte/mir vorstelle’, i.e. ‘the way I want/would like’) for the ideal state phrased ‘as I want’ in the original English tool as they seemed more suited to the respective specific contexts.

The *Control over daily life* domain could not easily be translated over. Thus, two options for an adaptation were tested with informal carers. The cognitive interviews for this domain revealed that option 1 ‘nach eigenem Ermessen’ (at their own discretion) was perceived as too broad a concept whereas option 2 ‘selbstbestimmt gestalten’ (being able to influence) was viewed as more specific and tangible. The second option was better understood and, therefore, used in the final questionnaire for the quantitative data collection.

The domains *Self-care*, *Social participation and involvement,* and *Occupation* were understood as intended. As interviewees preferred short names for ASCOT-Carer domains, the final wording for the *Social participation* *and involvement* domain was ‘Sozialleben’ (social life) instead of ‘Soziale Kontakte und Engagement im gesellschaftlichen Lebens’ (social contacts and social involvement). One of the response options for the *Occupation* domain was changed to ‘Ich verbringe etwas, aber nicht ausreichend Zeit’ instead of ‘Ich verbringe einige, aber nicht ausreichend Zeit’ (‘I do some of the things I value or enjoy with my time, but not enough’), as the German term ‘einige’ was understood to mean ‘a lot of’. The German translation of the questions about *Feeling supported and encouraged* was associated, as intended, with the feeling of appreciation and empathy from others, but also with financial support. Some respondents thought about having support in general (the fact that a care worker comes) while others reflected more on the actual support they were receiving by particular care workers or services. Informal carers, who were not in direct contact with care workers, seem to be less likely to feel supported by them and tended to think about support in more general terms. For one respondent, the idea of feeling supported as an informal carer did not seem relevant (this might be due to the fact that the respondent was also employed in the care sector and did not feel the need to rely on getting support).

*Personal safety* turned out not always to be understood in the same way. Some respondents did not find it easy to define or delimit the concept of personal safety and included financial security. In addition, some reflected on safety in connection with formal help. Feelings of better safety seemed to result from receiving regular formal help and from having trust in the care workers. On the other hand, some characteristics of service provision, such as frequent changes of care workers, seemed to negatively affect feelings of safety. While some respondents refer to injury risk and physical strain due to caregiving, others had difficulties to understand how safety is related to their caregiving and had a different idea of safety in mind than being safe from accidents and abuse. These different ways to interpret ‘personal safety’ stress the importance of using the interviewer prompt with this domain (‘with ‘feeling safe’ we mean feeling safe from fear of abuse, being attacked or other physical harm, such as accidents, which are *a result of your caring role*’).

The German translation of the questions about *Space and time to be yourself* captured, as intended, the carers’ ability to have enough time away from caring and to have a life of their own outside of the caring role. One respondent interpreted ‘space’ literally as having a separate apartment, not shared with the cared-for person, and thus having a life outside of the caring role.

Based on these findings, a few recommendations for amendments to the questionnaire were sent to the translation agency, which incorporated changes in consultation with the Austrian researchers and the ASCOT development team. The translated version (following proofreading and final checks) was then taken forward in the field phase (quantitative data collection).

## Results part 2

### Survey sample characteristics

In total, 344 informal carers completed the standardized personal interviews (which meets the recommended sample size of 100 and more respondents for hypothesis testing for construct validity [13]). Table 2 shows the sample characteristics: two thirds of the informal carers in the sample were women, just over half of the sample was the child of the cared-for person and 38% were the partner, all others were other family members and non-kin, such as friends or neighbors. Almost two thirds of the informal carer sample lived in the same household as the home care service user. About half of the informal carer sample supported their relatives or non-kin less than 5 years. Of the sample, 20% spent less than 10 hours per week in caregiving, 40% between 10 and 50 hours, 16% 50–99 hours and 20% more than 100 hours per week. In total, only one in four informal carers had taken up services *for* carers, such as information and advice, support from a carers’ group or short-term care. Details concerning the study design, data collection and extensive sample characteristics of the Austrian informal carer data are provided elsewhere [19].

**Table 2:** Sample characteristics: informal carers (Austria). *Source* WU, EXCELC INT C AUT 2016/2017, *n* = 344

	Frequency	%
Sex		
Female	233	67.7
Male	111	32.3
Relationship to person cared for		
Partner	132	38.4
Child	177	51.4
Other	34	9.9
Missing	1	0.3
Co-residence		
Yes	218	63.4
No	125	36.3
Missing	1	0.3
Duration of caring		
Less than a year	14	4.07
1 year, less than 3 years	74	21.51
3 years, less than 5 years	77	22.38
5 years, less than 10 years	90	26.16
More than 10 years	85	24.71
Missing	4	1.16
Informal care hours per week		
Less than 10 hours/week	67	19.5
10–49 hours/week	140	40.7
50–99 hours/week	55	16.0
100+ hours/week	69	20.1
Missing	13	3.8
Services for informal carers		
Yes	84	24.4
No	260	75.6
Total	344	100.0

Percentages may not total 100.0% due to rounding

The distribution of responses for each ASCOT-Carer domain and the distributional statistics for the overall ASCOT-Carer score are shown in Table 3. The percentage of missing values was generally low, ranging from 0.2% (Self-care) to 3.5% (Feeling supported and encouraged), indicating a good acceptance of the German version of the ASCOT-Carer instrument. For this reason, no imputation was performed when investigating item-level and overall validity. Individuals with missing values in at least one of the domains were therefore not considered in the validation of the total score, they were, however, included in domain-specific analyses for the domains in which they had given valid answers.

**Table 3:** Responses to the German ASCOT questionnaire for informal carers (German ASCOT-Carer). *Source* WU, EXCELC INT C AUT 2016/2017, *n* = 344

German ASCOT-Carer response options	Occupation Freq. (%)	Control Freq. (%)	Social participation Freq. (%)	Feeling supported Freq. (%)	Self-care Freq. (%)	Personal safety Freq. (%)	Space and time Freq. (%)
High needs	12 (3.49)	22 (6.40)	11 (3.20)	16 (4.65)	39 (11.34)	6 (1.74)	13 (3.78)
Some need	147 (42.73)	106 (30.81)	74 (21.51)	73 (21.22)	94 (27.33)	28 (8.14)	111 (32.27)
No needs	117 (34.01)	148 (43.02)	127 (36.92)	139 (40.41)	135 (39.24)	109 (31.69)	159 (46.22)
Ideal state	66 (19.19)	66 (19.19)	130 (37.79)	104 (30.23)	75 (21.80)	198 (57.56)	59 (17.15)
Missing	2 (0.58)	2 (0.58)	2 (0.58)	12 (3.49)	1 (0.23)	3 (0.87)	2 (0.58)

	Mean (SD)	Median	Min	Max	*n*
German ASCOT-Carer: index (range: 0–21)	13.45 (4.13)	14	2	21	328

Item response distributions can also be used to assess potential floor or ceiling effects, which would manifest themselves in particularly high cell counts in the highest or lowest categories. Generally, frequencies were highest in the middle categories except for the domains of *Social participation and involvement* and *Personal safety*, where most respondents were in the ‘ideal state’ category. As all domains are scaled according to a similar logic, high levels of ‘ideal state’ answers in those two categories are not seen as indication of a ceiling effect, but rather a reflection of the respondents’ situations in these specific areas of life.

### Construct validity of the German ASCOT-Carer

#### Construct validity of the overall ASCOT-Carer score (German version)

To evaluate construct validity, we first examined correlations of the ASCOT-Carer total score (LTC-QoL score) with related concepts (Table 4). Significant, albeit moderate, correlations [29] were found for all variables (EQ-5D index, overall QoL, CES and ZBI as well as weekly care hours) with the overall ASCOT-Carer score. As expected, the EQ-5D index (H3), QoL (H1) and CES (H2) were positively correlated with ASCOT-Carer score, suggesting that better health-related QoL (EQ-5D), overall QoL (7-point QoL scale) and carer’s experience (CES score) were associated with a higher LTC-QoL for carers. ZBI score (H4) was negatively correlated with the ASCOT-Carer score which suggests, as hypothesized, that negative experiences of caregiving (high ZBI-values, indicating, for example, being stressed and unsure) relate to low LTC-QoL for informal carers.

**Table 4:** Significance of correlations between ASCOT-Carer score and scores of comparator outcome measurement instruments. *Source* WU, EXCELC INT C AUT 2016/2017

Scales expected to be related	ASCOT-Carer score Spearman's rho (*p*-value)	*n*
EQ-5D index	0.382 (< 0.001)	324
QoL^a^	0.501 (< 0.001)	169
CES score	0.370 (< 0.001)	299
ZBI score	− 0.541 (< 0.001)	320

Thresholds according to Cohen [29]: 0.10 for small, 0.30 for medium and 0.50 for large effect sizes

^a^The seven-point QoL-scale was added to the questionnaire at a later stage in the field phase, hence the lower number of respondents

#### Domain-specific construct validation of the ASCOT-Carer (German version)

Table 5 summarize the results for scores of comparator outcome measures to be related to *all* seven ASCOT-Carer domains, state the significance and direction of the association. As expected, a number of instruments (QoL (h1), EQ-5D index (h2), CES (h3) and ZBI (h4)) scores were related to all ASCOT-Carer items. In general, associations were significant at the 1% level (see Table 5) for CES and ZBI as well as EQ-5D, the latter with the exception of *Occupation* (10%), *Self-Care* (5%) and *Space and time to be yourself* (not significant).

**Table 5:** EQ-5D, CES and ZBI scores by the German ASCOT-Carer domains. *Source* WU, EXCELC INT C AUT 2016/2017

ASCOT-Carer attributes per domain	QoL (*n* = 169)	EQ-5D (*n* = 338)	CES (*n* = 309)	ZBI (*n* = 333)
*Occupation*				
High needs	3.14 (7)	0.90 (12)	11.82 (11)	5.67 (12)
Some needs	3.41 (76)	0.84 (145)	12.75 (133)	7.07 (142)
No needs	4.07 (55)	0.89 (116)	14.11 (108)	4.89 (113)
Ideal state	4.13 (31)	0.90 (65)	14.35 (57)	3.36 (66)
*F* statistic and significance	10.06***	2.47*	8.77***	25.03***
Omega squared	0.14	0.01	0.07	0.18
*Control over daily life*				
High needs	3.27 (11)	0.83 (22)	11.32 (19)	7.00 (21)
Some needs	3.35 (54)	0.83 (104)	13.11 (96)	7.12 (101)
No needs	3.96 (70)	0.89 (146)	13.83 (137)	5.35 (146)
Ideal state	4.09 (34)	0.92 (66)	14.02 (57)	3.08 (65)
*F* statistic and significance	8.09***	4.49***	6.19***	25.19***
Omega squared	0.11	0.03	0.05	0.18
*Social participation and involvement*				
High needs	3.31 (42)^a^	0.76 (11)	10.55 (11)	8.91 (11)
Some needs		0.82 (72)	12.20 (66)	6.83 (70)
No needs	3.81 (70)	0.89 (127)	13.84 (115)	5.52 (123)
Ideal state	4 (56)	0.89 (128)	14.16 (117)	4.56 (129)
*F* statistic and significance	5.72***	4.84***	13.33***	11.83***
Omega squared	0.08	0.03	0.11	0.09
*Feeling supported and encouraged*				
High needs	3.71 (7)	0.81 (15)	10.27 (15)	7.06 (16)
Some needs	3.08 (37)	0.81 (73)	11.55 (64)	7.53 (72)
No needs	3.76 (71)	0.88 (138)	13.97 (128)	5.77 (133)
Ideal state	4.18 (51)	0.91 (102)	14.56 (96)	3.74 (103)
*F* statistic and significance	12.39**	5.15***	30.72***	23.92***
Omega squared	0.17	0.04	0.23	0.18
*Self-care (Looking after yourself)*				
High needs	2.86 (16)	0.80 (38)	11.22 (37)	7.41 (39)
Some needs	3.44 (48)	0.86 (94)	13.31 (85)	7.07 (89)
No needs	3.91 (68)	0.88 (134)	13.98 (120)	5.13 (131)
Ideal state	4.22 (37)	0.91 (73)	14.10 (68)	3.43 (75)
*F* statistic and significance	12.99***	3.4**	11.99***	25.23***
Omega squared	0.18	0.02	0.10	0.18
*Personal safety*				
High needs	3.19 (16)^a^	0.71 (6)	12.50 (6)	7.33 (6)
Some needs		0.71 (28)	11.85 (26)	7.93 (28)
No needs	3.50 (68)	0.86 (108)	12.98 (99)	6.10 (105)
Ideal state	4.05 (85)	0.90 (195)	14.03 (177)	4.81 (193)
*F* statistic and significance	11.32***	12.89***	7.11***	9.95***
Omega squared	0.10	0.10	0.06	0.07
*Space and time to be yourself*				
High needs	3.00 (6)	0.80 (13)	10.23 (13)	8.77 (13)
Some needs	3.43 (54)	0.86 (109)	12.93 (100)	7.17 (106)
No needs	3.94 (82)	0.88 (159)	13.97 (145)	4.95 (155)
Ideal state	3.96 (27)	0.90 (57)	14.04 (51)	3.47 (59)
*F* statistic and significance	5.72***	1.7 n.s	10.39***	26.35***
Omega squared	0.08		0.08	0.19

Thresholds according to Cohen [29]: 0.01 for small, 0.06 for medium and 0.14 for large effect sizes

*n.s.* not significant

***Significant at 1% level, **significant at 5% level, *significant at 10% level

^a^Lowest two levels (high needs and some needs) were collapsed for the analysis due to low cell counts

Tables 6 and 7 shows the results for single items of comparator outcome measures to be related to ASCOT-Carer domain and specific subgroups of informal carers expected to be related with the ASCOT-Carer domains.

**Table 6:** Relationship between the German ASCOT-Carer domains and related items of comparator outcome measures or subgroup characteristics (continued in Table 7). *Source* WU, EXCELC INT C AUT 2016/2017, Benjamini & Hochberg correction for multiple testing applied (ASCOT-Carer domain-wise)

ASCOT-Carer domains	Response level	Occupation^a^				Control over daily life^a^				Self-care (Looking after yourself)					Personal safety^a^			
		High/some needs	No needs	Ideal State	Sig.	High/some needs	No needs	Ideal State	Sig.	High needs	Some needs	No needs	Ideal State	Sig.	High/some needs	No needs	Ideal State	Sig.
	n	159	117	66		128	148	66		39	94	135	75		34	109	198	
*Well-being & health*																		
EQ-5D1—mobility	% no problems														55.88	74.31	82.83	***
EQ-5D2—self care	% no problems									**87.18**	**94.68**	**91.04**	**98.67**	**	85.29	89.91	96.45	**
EQ-5D3—usual activities	% no problems	**55.97**	**84.48**	**89.23**	***	56.25	76.71	92.42	***						41.18	66.97	80.1	***
EQ-5D4—pain	% no problems														17.65	31.48	56.06	***
EQ-5D5—depression	% no problems														35.29	53.21	73.23	***
*Burden of caring*																		
CES1—activities outside caring	% most	**15.19**	**57.26**	**83.33**	***													
CES2—support from family/friends	% most																	
CES3—support from organizations/government	% most																	
CES5—control over caring	% most					**83.46**	**78.08**	**74.60**	**ns**									
ZBI1—not enough time for themselves	% frequently/always	**34.81**	**9.40**	**4.55**	***					41.03	30.11	12.59	10.67	***				
ZBI2—stressed because of compatibility of caring and other responsibilities	% frequently/always					**34.40**	**20.27**	**7.58**	***									
ZBI4—feeling uncertain about SU	% sometimes/frequently/always					**44.44**	**25.85**	**12.31**	***									
Care hours per week	% 50 h or more	52.26	26.32	20.00	***	57.72	25.69	24.19	***	47.37	47.25	35.66	22.54	***	55.88	40.57	32.09	**
SU’s cognitive skills	% severely impaired	43.04	31.30	20.00	***					51.28	44.09	34.33	15.07	***	44.12	43.52	28.72	**
SU’s challenging behavior	% sometimes/frequently	34.81	28.21	16.67	**					43.59	31.18	29.63	17.57	**	38.24	38.53	21.94	***

Comparisons with other outcome measures (convergent validity) are printed in bold; else, comparisons with subgroups of informal carers (discriminative validity)

*ns* not significant

***Significant at 1% level, **significant at 5% level

^a^Lowest two levels of the domain were collapsed due to small cell counts; SU…care service user

**Table 7:** Relationship between German ASCOT-Carer domains and items of comparator outcome measures or subgroup characteristics (continued from Table 6). *Source* WU, EXCELC INT C AUT 2016/2017, Benjamini & Hochberg correction for multiple testing applied (ASCOT-Carer domain-wise)

ASCOT-Carer domains	Response levels	Social participation and involvement^a^				Space and time to be yourself^a^				Feeling supported and encouraged^a^			
		High/some needs	No needs	Ideal State	Sign	High/some needs	No needs	Ideal State	Sign	High/some needs	No needs	Ideal State	Sign
	n	85	127	130		124	159	59		89	139	104	
*Burden of caring*													
CES1—Activities outside caring	% most					**13.01**	**51.57**	**81.36**	***				
CES2—support from family/friends	% most									**17.98**	**51.08**	**66.02**	***
CES3—support from organizations/government	% most									**4.60**	**21.80**	**34.62**	***
CES5—control over caring	% most												
ZBI1—not enough time for themselves	% frequently/always					**38.71**	**10.13**	**8.47**	***				
ZBI2—stressed because of compatibility of caring and other responsibilities	% frequently/always	36.14	20.63	16.92	***								
ZBI4—feeling uncertain about SU	% frequently/always									**16.85**	**5.88**	**5.83**	***
Care hours per week	% 50 h or more	51.25	40.00	25.20	***	52.10	29.68	27.27	***				
Breaks from caring (2 days)	% yes					55.65	73.58	77.59	***				
*Social contact*													
Speak to relatives/friends	% weekly	**78.82**	**81.10**	**91.54**	**								
Speak to neighbors	% weekly	**57.14**	**77.78**	**74.42**	***								
Meet up with relatives/friends	% weekly	**32.94**	**55.91**	**77.69**	***								
*Process quality (carer’s perspective)*													
Carer's satisfaction with LTC services	% (extremely) satisfied									**86.05**	**94.74**	**94.85**	**

Comparisons with other outcome measures (convergent validity) are printed in bold; else, comparisons with subgroups of informal carers (discriminative validity)

*ns* not significant

***Significant at 1% level, ** significant at 5% level

^a^Lowest two levels of attribute are collapsed because of small numbers

#### Occupation

As expected, the EQ-5D item ‘usual activities’ (h-occu1) and the CES item ‘life outside caring’ (h-occu2) were significantly positively, and the ZBI item ‘time for oneself’ (h-occu3) negatively associated with the ASCOT-Carer item *Occupation*, which aims to capture meaningful and enjoyable activities. In addition, associations between *Occupation* and characteristics of the care setting, namely challenging behavior (h-occu5) and cognitive skills of the cared-for person (h-occu6) and care hours (h-occu4) were associated in the expected direction at the 5% (challenging behavior) or 1% level, suggesting that challenging behavior and low cognitive skills as well as many weekly care hours were related to low QoL in the domain *Occupation* of the German ASCOT-Carer (Tables 6 and 7).

#### Control over daily life

In line with the results of the validation for the English original [3], we found a significant positive association between the EQ-5D item ‘usual activities’ and the ASCOT-Carer item *Control over daily life* (h-cont1). As expected, the ZBI items ‘stressed because of compatibility of caring and other responsibilities’ (h-cont3) and ‘feeling uncertain about service user’ (h-cont4) were negatively associated with *Control*. The CES item ‘control over caring’, however, was not found to be positively associated (h-cont2, not confirmed). We found a highly significant negative association between the *Control* item and weekly care hours (h-cont5), with higher care hours per week being related with lower feelings of being in control of daily life (Tables 6 and 7).

#### Self-care (Looking after yourself)

As hypothesized, the EQ-5D item ‘self-care’ (h-care1) was significantly positively at the 5% level, and ZBI ‘time for oneself’ (h-care2) negatively at the 1% level related to the ASCOT-Carer *Self-care (Looking after yourself)* domain. The ASCOT-Carer item *Self-care* was associated negatively with both the service user’s cognitive performance (h-care4) and challenging behavior (h-care5) and care hours (h-care3), as expected (Tables 6 and 7).

#### Personal safety

All five EQ-5D items were significantly positively associated with the *Personal safety* domain (h-safe1). Also, in line with the hypotheses, we found significant negative associations between the ASCOT-Carer item and both service user cognitive skills (h-safe3), challenging behavior of the cared-for person (h-safe4) and care hours (h-safe2). As expected, informal carers with no problems in any of the EQ-5D-items, those whose cared-for person had no cognitive impairments or showed no challenging behavior, as well as informal carers who helped less than 50 hours a week, reported better *Personal safety* (Tables 6 and 7).

#### Social participation and involvement

Tables 6 and 7 show that, as expected, the ZBI item ‘stressed because of compatibility of caring and other responsibilities’ (h-soci1) as well as the number of weekly care hours (h-soci2) were each negatively related to the ASCOT-Carer *Social participation and involvement* domain. In line with the hypothesis, the social contact variables were also found to be positively related at the 1% level (‘speak to neighbors’, ‘meet up with relatives/friends’) and 5% level (‘speak to relatives/friends) (h-soci3).

#### Space and time to be yourself

As expected, we found a significant positive association between the CES item ‘activities outside caring’ (h-time1) and the ASCOT-Carer item *Space and time to be yourself.* Negative associations were found between each of the variables (the ZBI item ‘time for oneself’ (h-time2) and care hours (h-time3)), while the possibility of taking breaks from caring and the ASCOT-Carer domain *Space and time to be yourself* were positively related (h-time4).

#### Feeling supported and encouraged

The ASCOT-Carer domain *Feeling supported and encouraged* was positively associated with the CES items ‘support from family/friends’ (h-supp1) and ‘support from formal services’ (h-supp2). We found the ZBI item ‘feeling uncertain about the person cared for’ (h-supp3) to be negatively related (significant at the 1% level) with the ASCOT-Carer domain *Feeling supported and encouraged*. Finally, the process quality variable (overall satisfaction with LTC services) was also significantly associated with ASCOT-Carer *Feeling supported* *and encouraged* (see Tables 6 and 7).

## Discussion

The translation and cultural adaptation according to ISPOR TCA guidelines aimed to develop a German version of the ASCOT-Carer instruments (INT4, SCT4) that has sufficient linguistic, content and construct validity and can be employed in German-speaking surveys as a measure of LTC-QoL of informal carers.

The analysis of cognitive debriefing interviews provided evidence for linguistic and content validity. The interviews showed no major issues with comprehension of the German translation of ASCOT-Carer, except for the *Personal safety* domain, where the restriction to ‘as a result of caring role’ was not always picked up by the informal carers. Therefore, to ensure the understanding of the *Personal safety* domain, we recommend briefing interviewers to pay extra attention when addressing the domain and to stress the importance of the prompt included in the question.

In addition, we found solid evidence to support convergent validity as part of construct validity of the translated German ASCOT-Carer. The ASCOT-Carer score was significantly correlated with other measures of conceptually-related constructs, particularly to scales also capturing carers’ quality of life (such as EQ-5D index and measures of carers’ experience and burden). As to be expected, the weakest association was found with the EQ-5D index that seeks to capture health-care instead of long-term care-related QoL.

For most of the ASCOT-Carer domains, significant associations with conceptually related constructs were found, except for *Personal Safety*, with no corresponding alternate measure in the data, and for the *Control over daily life* domain and the CES item ‘control over caring’, which seem to measure different aspects of having control. The ASCOT-Carer domain *Control over daily life* was intended to reflect a broader concept that may also be influenced by other areas of life, not only caring [34], while the CES item has a narrower, more specific focus.

The comparison between subgroups of informal carers to explore discriminative or known-group validity focused on characteristics that are well supported by previous studies. As expected, informal carers with high care intensity or no opportunity to take a break from caring showed lower LTC-QoL, as informal carers who cared for service users with low cognitive skills and challenging behavior.

While the analyses presented in this paper supported the construct validity of a culturally adapted German version of ASCOT-Carer and may be useful as a reference for assessing the validity of ASCOT-Carer translated to other languages, there are some limitations to this study. To begin with, we did not match English and Austrian samples of informal carers to investigate cross-cultural validity as defined by COSMIN study design checklist [13]. Second, we did not investigate measurement properties that require standardized interview data at two time points (e.g. test–retest reliability and responsiveness, such as the sensitivity of the instrument to changes of LTC service receipt over time) as this was beyond the financial means of this project. We thus encourage future research to assess these measurement properties.

The findings of this study provided good evidence for a culturally adapted German version of the ASCOT-Carer instrument. The cognitive debriefing interviews support its linguistic and content validity. Since almost all related constructs were significantly associated with the German ASCOT-Carer (score and individual domains) and in the expected direction (convergent validity), and since the same holds true sub-groups of informal carers (discriminative or known-group validity), there is good evidence for its construct validity. Furthermore, the analysis of qualitative as well as quantitative data comes to similar conclusions as reported for the original English ASCOT-Carer instrument [2, 3] and therefore support the construct validity of the German translation. These findings support the use of the German ASCOT-Carer instrument to capture LTC-related QoL for informal carers in Austria and other German speaking countries and can thus be utilized for national evaluations of LTC outcomes and comparative studies. Research is encouraged to assess further measurement properties of the translated instrument.

## Electronic supplementary material

Below is the link to the electronic supplementary material.

Supplementary file1 (DOCX 211 kb)

Supplementary file2 (DOCX 26 kb)

## Footnotes

1 https://www.pssru.ac.uk/ascot/tools/.

2 https://www.pssru.ac.uk/ascot/translations/ or https://short.wu.ac.at/ascot.

3 https://www.qualtrics.com/de/.
