# Supplementary material for: Translation, cultural adaptation and construct validity of the German version of the Adult Social Care Outcomes Toolkit for informal Carers (German ASCOT-Carer)
Source: Qual Life Res. 2020 Nov 2;30(3):905–20. doi: 10.1007/s11136-020-02682-4 (PMC7952350; doi:10.1007/s11136-020-02682-4)
Supplement: Supplementary file 1 — Supplementary file1 (DOCX 211 kb) [file 11136_2020_2682_MOESM1_ESM.docx]

Supplementary material to the manuscript: **Translation and Cross-Cultural Adaptation of the German Version of the Adult Social Care Outcomes Toolkit for Informal Carers (German ASCOT-Carer)**

Quality of Life Research: QURE-D-19-01475

As stated in the background section of the paper, assessing structural validity by means of performing factor analysis or assessing internal consistency is not relevant for instruments following a formative measurement model, such as the ASCOT-Carer measure. This is in line with the COSMIN checklist’s recommendations regarding patient-reported outcome measurement instruments [1]. For these reasons, we have not looked into structural validity and internal consistency as part of the paper. However, we acknowledge that the factor structure of an instrument provides potentially useful information for researchers, regardless of its relevance for instrument validation. For this reason, and due to the editor´s request, we provide a brief overview of results from a confirmatory factor analysis of the German version of the ASCOT-Carer.

*Summary*

We conducted a confirmatory factor analysis (CFA) and calculated Cronbach´s alpha using the Austrian data and compared them to analyses published for the English ASCOT-Carer [2]. The results are as expected, with the English and the German version performing similarly. We report here key findings, but do not touch upon internal consistency or structural validity as they are not relevant for the underlying measurement model of the ASCOT-Carer.

*Comparison between the English and German version of the ASCOT-Carer instrument: Cronbach´s alpha and CFA*

Cronbach’s alpha for the German version of the ASCOT-Carer was 0.84, compared to 0.87 for the English original. CFA shows that the seven domains of the four-level ASCOT-Carer instrument loaded onto a common factor for both the English and the German version.

Overall, the fit between the one-factor model and the data was slightly better in the Austrian sample, as shown by the comparison of fit indices (Table 1). All domains loaded significantly onto the factor at the 1% level, with loadings ranging between 0.47 and 0.79 for the Austrian sample (Figure 2a). Factor loadings for the English version were very similar and ranged between 0.47 and 0.84 (Figure 2b). In both versions, the domains ‘Personal safety’ and ‘Feeling supported and encouraged’ had the lowest factor loadings. For the English version, the domains ‘Occupation’ and ‘Control over daily life’ had the highest loadings, for the German version, ‘Space and time to be yourself’ and ‘Occupation’. Overall, these results indicate that the factor structures of the original and the translated measure are fairly comparable, even though model fit for the one-factor model was better in the Austrian data.

*Figures and Tables*

|  | adequate model fit | Austrian Sample | English Sample |
| --- | --- | --- | --- |
| n |  | 328 | 387  52.55  14  <0.001  0.085  0.037  0.969  0.953  0.901 |
| chi2 |  | 21.18 |  |
| df |  | 14 |  |
| p-value | >0.05 | 0.097 |  |
| RMSEA | <0.06 | 0.04 |  |
| SRMR | <0.08 | 0.028 |  |
| CFI | >0.95 | 0.990 |  |
| TLI | >0.95 | 0.986 |  |
| CD |  | 0.864 |  |
| Source: WU, EXCELC INT C AUT 2016/17; [2] | | | |

Figure 1. Scree Plot: German version of ASCOT-Carer Table 1. CFA – One-factor models of ASCOT-Carer

Source: WU, EXCELC INT C AUT 2016/2017 n=328

Figure 2a. Standardized parameter estimates for the one-factor structure of the seven ASCOT-Carer domains of the German version

0.75

0.7

0.73

0.47

0.65

0.79

0.48

Source: WU, EXCELC INT C AUT 2016/2017 n=328

Figure 2b. Standardized parameter estimates for the one-factor structure of the seven ASCOT-Carer domains of the English version


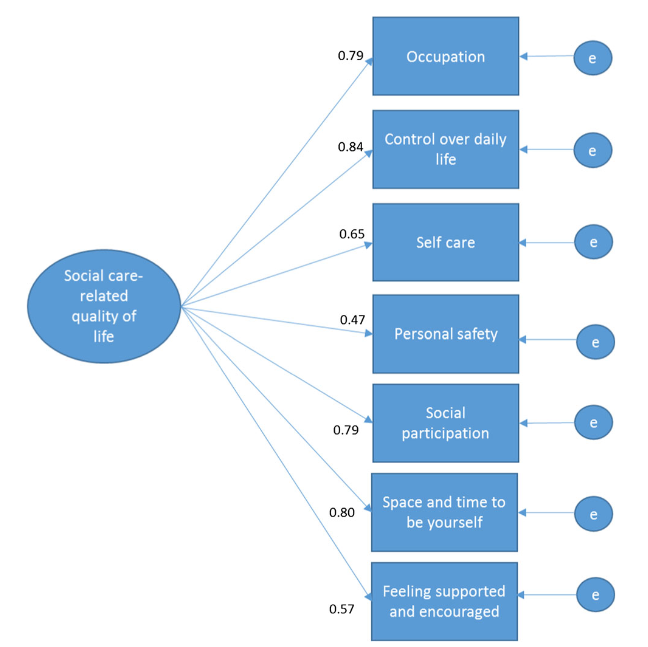


Note: Self care = Looking after yourself

Source: [2]

*References*

1. Mokkink, L. B., Prinsen, C. A., Patrick, D. L., Alonso, J., Bouter, L. M., De Vet, H., & Terwee, C. B. (2019). COSMIN Study Design checklist for Patient-reported outcome measurement instruments.

2. Rand, S. E., Malley, J. N., Netten, A. P., & Forder, J. E. (2015). Factor structure and construct validity of the Adult Social Care Outcomes Toolkit for Carers (ASCOT-Carer). Quality of Life Research, 24(11), 2601-2614.
