# Supplementary material for: Translation, cultural adaptation and construct validity of the German version of the Adult Social Care Outcomes Toolkit for informal Carers (German ASCOT-Carer)
Source: Qual Life Res. 2020 Nov 2;30(3):905–20. doi: 10.1007/s11136-020-02682-4 (PMC7952350; doi:10.1007/s11136-020-02682-4)
Supplement: Supplementary file 2 — Supplementary file2 (DOCX 26 kb) [file 11136_2020_2682_MOESM2_ESM.docx]

# Appendix

Table 8: Comparative outcome measures and characteristics of subgroups of informal carers expected to be associated with the German ASCOT-Carer scores or domains

| **Variables** | **Hypotheses** | **Expected** **association with ASCOT-Carer score or domains** |
| --- | --- | --- |
| **Well-Being & Health** |  |  |
| Self-perceived QoL | H1  h1 | low to moderate positive correlation between the *ASCOT-Carer score* and self-rated QoL [4; 35]  positive associations of varying degrees between QoL and *all ASCOT-Carer domains* [4; 35] |
| EQ-5D | H3  h2  h-occu1  h-cont1  h-care1  h-safe1 | low to moderate moderate positive correlation of the EQ-5D index with *ASCOT-Carer score* since LTC-QoL and HRQoL can be seen as partly overlapping concepts [4; 36; 37]  positive associations of varying degrees between the EQ-5D index and *all ASCOT-Carer domains* [4]  positive association between EQ-5D item usual activities and ASCOT-Carer *Occupation*, as performing usual activities is related to being able to do things one enjoys  positive associations between EQ-5D item usual activities and ASCOT-Carer *Control over daily life* as being able to perform those tasks could lead to higher perceived control over daily life  positive association between EQ-5D item self-care and ASCOT-Carer *Self-care,* as self-care (washing and dressing) is related to feelings of personal cleanliness  positive associations between all EQ-5D items (mobility, self-care, usual activities, pain, and anxiety/depression) and ASCOT-Carer *Personal safety* as they capture factors making a person to feel unsafe in their caring role. |
| **Burden of Caring** |  |  |
| Carer Experience Scale (CES) | H2  h3  h-occu2  h-cont2  h-time1  h-supp1  h-supp2 | low to moderate positive correlation with total *ASCOT-Carer score* [4]  positive associations of varying degrees between the CES score (carer experience) and *all ASCOT-Carer domains* [4]  positive association between CES1 (activities outside caring) and ASCOT-Carer *Occupation* [3]  positive association between CES5 (control over caring) and ASCOT-Carer *Control over daily life*  positive association between CES1 (activities outside caring) and ASCOT-Carer *Time and space* since it captures the freedom for carers to have a life outside of caring [3]  positive association between CES2 (support from family) and ASCOT-Carer *Feeling supported*  positive association between CES3 (support from formal services) and ASCOT-Carer *Feeling supported* |
| Zarit Burden Index (ZBI): screening version | H4  h4  h-occu3  h-cont3  h-cont4  h-care2  h-soci1  h-time2  h-supp3 | low to moderate negative correlation with *ASCOT-Carer score*  negative associations of varying degrees between the ZBI score (caregiver self-report measure) and *all ASCOT-Carer domains*  negative association between ZBI1 (time for oneself) and ASCOT-Carer *Occupation*  negative association between ZBI2 (stress between caring and other responsibilities) and ASCOT-Carer *Control* *over daily life* [3]  negative association between ZBI4 (feeling uncertain) and ASCOT-Carer *Control* *over daily life* [3]  negative association between ZBI1 (time for oneself) and ASCOT-Carer *Self-care*  negative association between ZBI2 (stress between caring and other responsibilities) and ASCOT-Carer *Social participation*  ZBI1 (time for oneself): carers with less time for themselves are expected to report lower levels in ASCOT-Carer *Time and* *space* [35; 38]  negative association between ZBI4 (feeling uncertain) and ASCOT-Carer *Feeling supported* [3]. |
| Care hours | h-occu4  h-cont5  h-care3  h-safe2  h-soci2  h-time3 | A high amount of care hours signify a burden on both their time and emotions as well as a disruption of daily life and the challenge of balancing care tasks and hours with being able to choose activities that meet the carer’s own needs/preferences [3; 39; 40].  to be negatively related with ASCOT-Carer *Occupation*  to be negatively related with ASCOT-Carer *Control*  to be negatively related with ASCOT-Carer *Self-care*  to be negatively related with ASCOT-Carer *Safety*  to be negatively related with ASCOT-Carer *Social participation*  to be negatively related with ASCOT-Carer *Time and space* |
| Breaks for carers | h-time4 | the possibility of having breaks is expected to be positively related with ASCOT-Carer *Time and space* [3]. |
| Home care service user’s cognitive performance  Home care service user displaying challenging behavior | h-occu5  h-care4  h-safe3  h-occu6  h-care5  h-safe4 | negative association with ASCOT-Carer *Occupation*  negative association with and ASCOT-Carer *Self-care* [40]  negative association with ASCOT-Carer *Personal* *safety* [41-43]  negative association with ASCOT-Carer *Occupation*  negative association with and ASCOT-Carer *Self-care* [40]  negative association with ASCOT-Carer *Personal* *safety* [41-43] |
| **Social Contact/Support** |  | Participating in social activities is associated with a higher QoL and lower reported burden among carers [35] . Therefore, contact with people outside of the home is expected to have a positive association with ASCOT-Carer *Social participation [4]*. |
| Speak to relatives/ friends on phone  Speak to neighbors  Meet up with relatives/friends | h-soci3  h-soci4  h-soci5 | positive association with ASCOT-Carer *Social participation*  positive association with ASCOT-Carer *Social participation*  positive association with ASCOT-Carer *Social participation* |
| **Process Quality** |  |  |
| Overall satisfaction with services | h-supp4 | Since ASCOT-Carer was developed to measure aspects of QoL targeted by LTC services and care support, a lower rating of satisfaction with the care provided by care workers is expected to be related to lower levels in the ASCOT-Carer *Feeling supported* domain, especially those items capturing coordination between/with services [44]. |

Notes: H indicates hypotheses related to the ASCOT score; h indicates hypotheses related to *all* ASCOT items, and h-clean/occu/soci etc. indicates hypotheses related to a specific ASCOT domain, such as personal cleanliness, occupation, social participation etc.

**References**

35. Mockford, C., Jenkinson, C., & Fitzpatrick, R. (2006). A review: carers, MND and service provision. Amyotrophic Lateral Sclerosis, 7(3), 132-141.

36. Rand, S., Malley, J., Towers, A.-M., Netten, A., & Forder, J. (2017). Validity and test-retest reliability of the self-completion adult social care outcomes toolkit (ASCOT-SCT4) with adults with long-term physical, sensory and mental health conditions in England. Health and Quality of Life Outcomes, 15(163).

37. Malley, J., Towers, A.-M., Netten, A., Brazier, J., Forder, J., & Flynn, T. (2012). An assessment of the construct validity of the ASCOT measure of social care-related quality of life with older people. Health and Quality of Life Outcomes, 10(21), 1-14.

38. Greenwood, N., Mackenzie, A., Cloud, G. C., & Wilson, N. (2009). Informal primary carers of stroke survivors living at home-challenges, satisfactions and coping: a systematic review of qualitative studies. Disability and Rehabilitation, 31(5), 337-351.

39. Glozman, J. M. (2004). Quality of life of caregivers. Neuropsychology review, 14(4), 183-196.

40. Morley, D., Dummett, S., Peters, M., Kelly, L., Hewitson, P., Dawson, J., Fitzpatrick, R., & Jenkinson, C. (2012). Factors Influencing Quality of Life in Caregivers of People with Parkinson's Disease and Implications for Clinical Guidelines. Parkinson's disease, 2012.

41. Nicholson, K. A. (2009). Carers' narratives: Finding dementia with Lewy bodies experiences. Australasian Journal on Ageing, 28(4), 177-181.

42. Reed, S. I. (2008). First‐episode psychosis: A literature review. International Journal of Mental Health Nursing, 17(2), 85-91.

43. Zegwaard, M. I., Aartsen, M. J., Cuijpers, P., & Grypdonck, M. H. F. (2011). A conceptual model of perceived burden of informal caregivers for older persons with a severe functional psychiatric syndrome and concomitant problematic behaviour. Journal of Clinical Nursing, 20(15‐16), 2233-2258.

44. Kang, X., Li, Z., & Nolan, M. T. (2011). Informal caregivers' experiences of caring for patients with chronic heart failure: systematic review and metasynthesis of qualitative studies. Journal of Cardiovascular Nursing, 26(5), 386-394.
